# Tension Pneumocephalus Following Bilateral Craniotomies

**DOI:** 10.7759/cureus.1358

**Published:** 2017-06-15

**Authors:** Fionn Coughlan, Alexander Lam, Stephen Honeybul

**Affiliations:** 1 Department of Neurosurgery, Royal Perth Hospital, Western Australia

**Keywords:** pneumocephalus, tension pneumocephalus, craniotomy, mount fuji sign, neurosurgery, neurotrauma, neuroradiology, radiology, ct, trauma

## Abstract

Pneumocephalus is defined as a collection of air within the cranial cavity. It is a common finding following head injury with skull base fracture and neurosurgical procedures. Tension pneumocephalus is a rare complication. The diagnosis is clinical and radiological with the characteristic Mount Fuji sign seen on computed tomography (CT). It is a neurosurgical emergency, and early recognition and treatment are vital.

## Introduction

Pneumocephalus is defined as a collection of air within the cranial cavity [1]. It is a common finding following craniotomy, burr-holes, and traumatic brain injury when there has been a skull base fracture [2-3]. In most instances, the air is resorbed over time and most patients are asymptomatic. A rare complication is that of tension pneumocephalus, which can lead to deterioration due to mass effect. It is a neurosurgical emergency and in most cases, it requires surgical intervention.

The most common radiological finding is that of the Mount Fuji sign [4-5]. This was first reported by Ishiwata, et al. in 1988 and demonstrates the pressure exerted on the frontal lobes [6]. The peaking sign is seen when a large collection of air over the anterior and lateral portion of the frontal lobes causes compression. This differs from the Mount Fuji sign in that the air does not cause separation of the tips of the frontal lobe. 

## Case presentation

**Figure 1:**
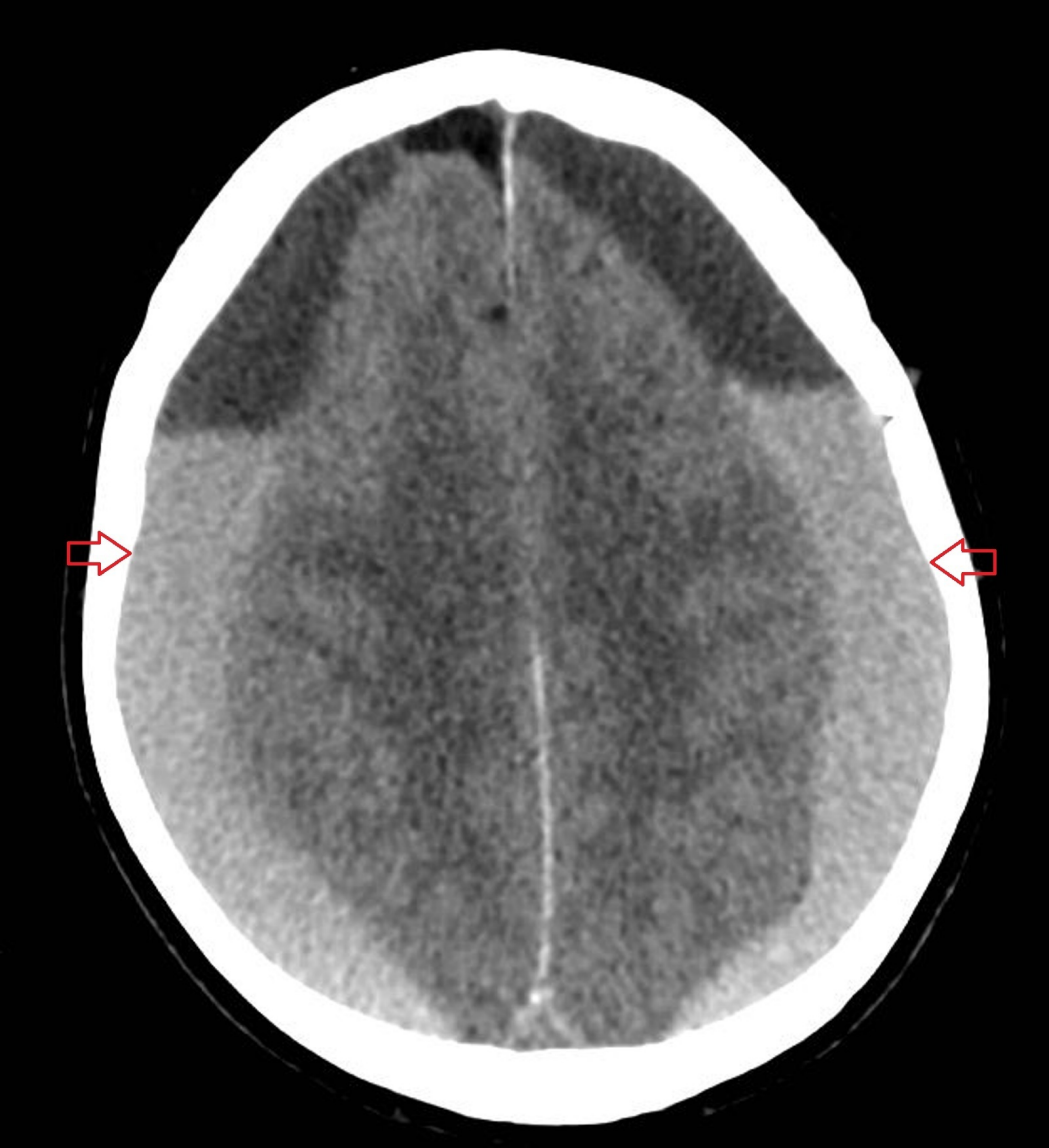
Axial non-contrast computed tomography scan of the brain Showing bilateral acute-on-chronic subdural haemorrhages.

A 70-year-old male initially presented to the local hospital following an alleged assault in which he sustained a minor head injury. The Glasgow coma score (GCS) on presentation was 15. Initial computed tomography (CT) scan of his brain was unremarkable. He was discharged but developed worsening headache and left-sided weakness one month later. He represented to the hospital and a repeat CT showed bilateral acute on chronic subdural haemorrhages (Figure 1).

**Figure 2:**
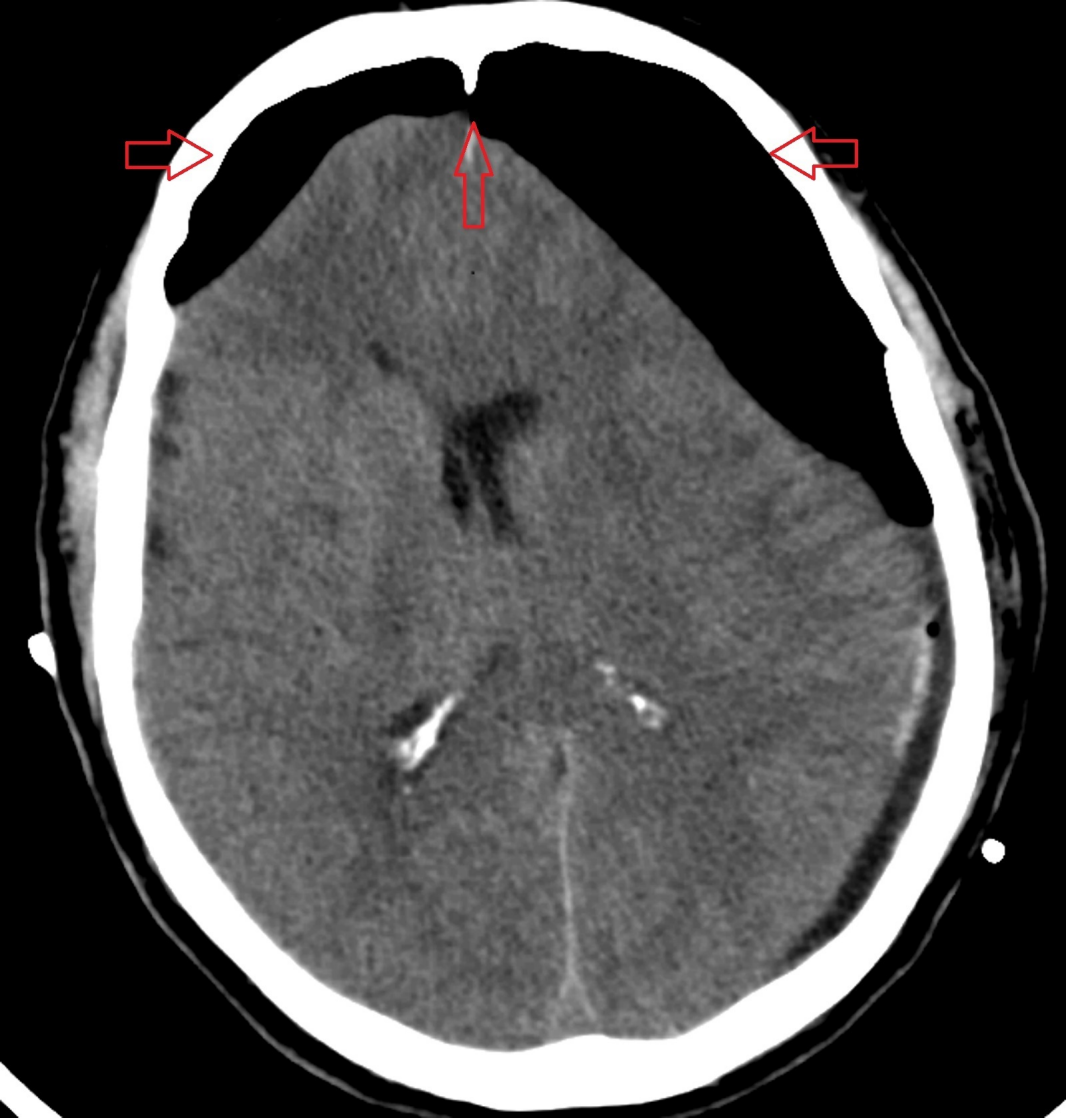
Axial non-contrast computed tomography scan of the brain Demonstrating tension pneumocephalus and the Mount Fuji sign.

The patient was taken to the theatre for bilateral craniotomies and evacuation. The dura was closed in a non-watertight fashion. Post-operatively he failed to recover. The GCS was three and his pupils were non-reactive to light. He was taken for an emergency CT brain scan which showed tension pneumocephalus with the presence of the Mount Fuji sign (Figure 2). He was immediately taken back to the theatre for a re-exploration of his left-sided craniotomy. The left wound was re-opened and the frontal lobe was noted to be under pressure and was decompressed. Warm saline was used to fill the cavity and the dura was closed to give a water-tight seal. A drain was placed and the craniotomy wound was closed.

**Figure 3:**
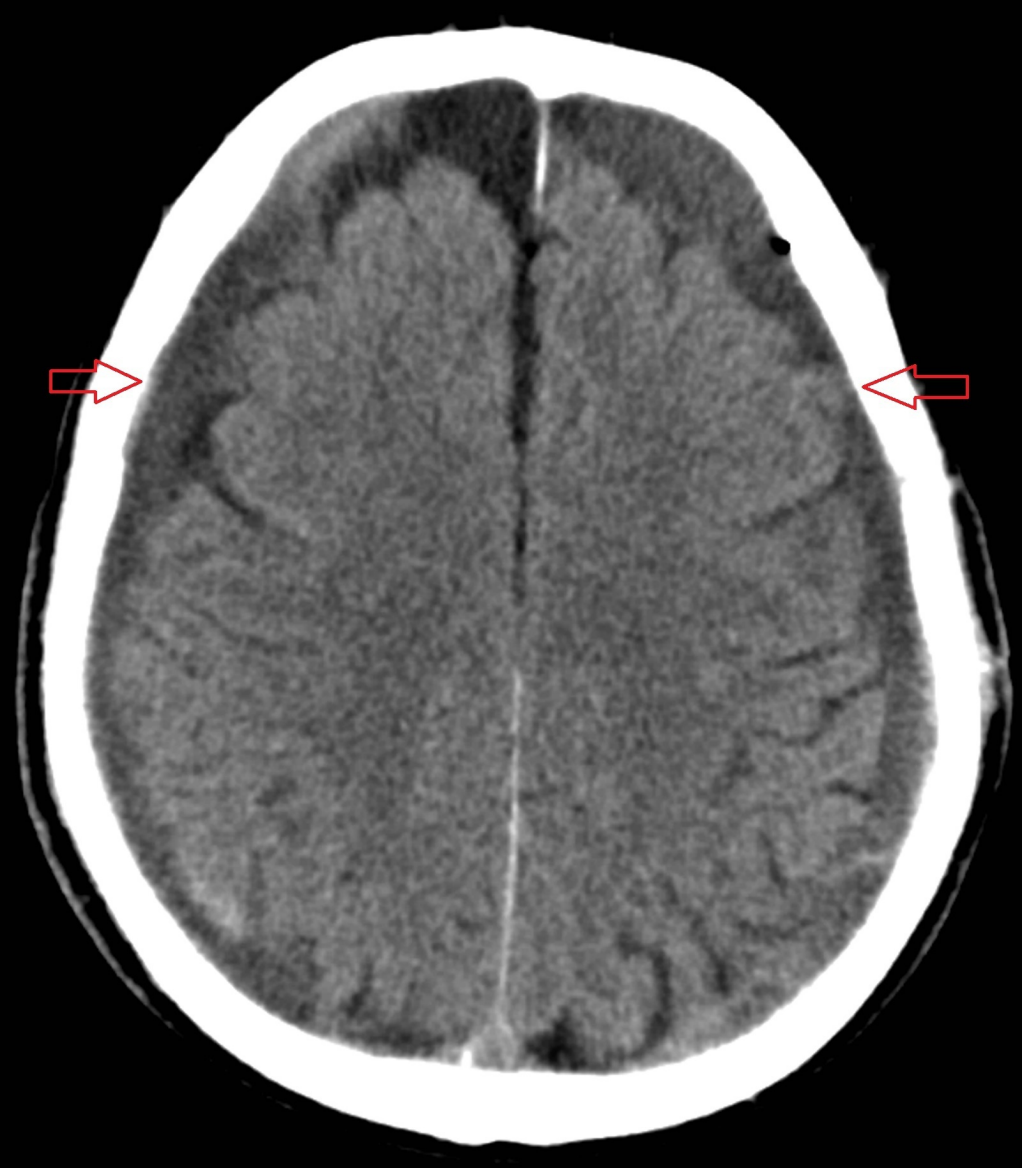
Axial non-contrast computed tomography scan of the brain Demonstrating resolution of the tension pneumocephalus and decompression of the brain.

He was extubated and transferred to the ward the following day. A full five-out-of-five power and GCS of 15 had returned to his left side. A repeat CT brain non-contrast showed resolution of the tension pneumocephalus and improvement in the bilateral subdural haematomas. Recovery was otherwise uneventful. Repeat CT performed two weeks post-operation showed improvement of the pneumocephalus and reduction in the size of the subdural haemorrhages (Figure 3). He was reviewed again at six weeks and at twelve weeks post-operation.

## Discussion

All patients have the potential to develop pneumocephalus following neurosurgical procedures or head injuries involving skull base fractures. Tension pneumocephalus is, however, rare. The incidence of tension pneumocephalus occuring, following evacuation of a chronic subdural haematoma, is reported to be 2.5% [6].

There are two postulated theories for this condition. The first is the ball-and-valve theory with air flowing in only one direction in the cranial cavity. This was described by Dandy, et al. The atmospheric to cranial pressure gradient is decreased leading to tamponade of the osteomeningeal fistula by the brain tissue, thus trapping intracranial air and causing a mass effect and midline shift [7].

The second theory is that of the ‘inverted-soda-bottle-effect’. This was described by Horowitz. Negative intracranial pressure, as can be caused by neurosurgical intervention or a Valsalva manoeuvre, can force air into the intracranial cavity when a fracture or fistula exists [8].

A clinical and radiological diagnosis with CT is the most commonly used modality to confirm the diagnosis. The symptoms of tension pneumocephalus are due to mass effect and can include reduced consciousness, restlessness, or focal neurological deficits. The CT differentiates intracranial air from denser substances such as fluid, brain tissue, and bone. The Mount Fuji sign is seen in cases of tension pneumocephalus and it indicates the necessity of emergent decompression. This sign is present when air compresses the frontal lobes, leading to the characteristic tented appearance. It results due to greater pressure of air present in the skull than the surface tension of cerebral fluid between the frontal lobes.

Tension pneumocephalus requires immediate treatment in majority of the cases [9]. In this case, early recognition allowed for a prompt return to the theatre for surgical decompression.

## Conclusions

Pneumocephalus is a common finding post craniotomy, burr hole, and skull base fracture. Tension pneumocephalus is a rare complication and is a neurosurgical emergency due to mass effect. It is important to recognise this condition so that prompt treatment can be initiated. The diagnosis is confirmed on CT scan with the typical findings of a Mount Fuji sign as described. Urgent treatment is required in most cases.

This case demonstrated the aetiology, symptoms, and radiological findings of tension pneumocephalus. The complication was recognised promptly and the correct treatment was initiated. The patient went on to have an uneventful recovery with resolution of the pneumocephalus.
